# Predictors of lung cancer patients’ behavioral intention to use virtual reality for treatment preparedness and shared decision-making: a Unified Theory of Acceptance and Use of Technology-based perception study

**DOI:** 10.1093/jamiaopen/ooag178

**Published:** 2026-09-25

**Authors:** Safa Elkefi, Steven K Feiner, Alicia K Matthews, Luis M Rocha, Carlos Gershenson-Garcia, Roberta Scheinmann

**Affiliations:** School of System Science & Industrial Engineering, Watson College of Engineering, Binghamton University, Binghamton, NY 13850, United States; Department of Computer Science, Columbia University, New York, NY 10027, United States; College of Nursing, RUSH University, Chicago, IL 60612, United States; School of System Science & Industrial Engineering, Watson College of Engineering, Binghamton University, Binghamton, NY 13850, United States; Católica Biomedical Research Center, Universidade Católica Portuguesa, Portugal; School of System Science & Industrial Engineering, Watson College of Engineering, Binghamton University, Binghamton, NY 13850, United States; School of Nursing, Columbia University, New York, NY 10032, United States

**Keywords:** virtual reality, lung cancer, technology acceptance model, oncology, digital health

## Abstract

**Objectives:**

We examined the intention of lung cancer patients to use virtual reality (VR) to support shared decision-making and treatment preparedness and identified predictors of this intention using the Unified Theory of Acceptance and Use of Technology (UTAUT).

**Methods:**

Two hundred cancer patients completed a survey. Logistic regression was used to identify the predictors of VR use for decision-making.

**Results:**

89.5% were willing to use VR; willing participants were younger than those who were not willing (60.8 ± 15.3 years; *P* < .001). Digital literacy was high in 89%, whereas health literacy was marginal/limited in 85%. Willingness was associated with greater technology use (*P* = .009). Performance expectancy strongly predicted intention: motivation to engage (Yes OR = 6.93; 95% CI, [4.06, 11.84]) and gaining knowledge (Yes OR = 15.20; 95% CI, [4.39, 52.56]); all *P* < .001. Effort expectancy (ease of navigation) was significant (Easy OR = 7.16; 95% CI, [2.50, 20.49]; *P* < .001), while mental/physical workload were not (*P* = .312/.732). Social influence from family/friends (OR = 3.38; 95% CI, [1.29, 8.84]; *P* = .016) and valuing family involvement (OR = 3.30; 95% CI, [1.32, 8.22]; *P* = .015) predicted intention; clinician encouragement did not (*P* = .283). Facilitating conditions were robust: peers also using VR (OR = 4.23; 95% CI, [1.46, 12.26]; *P* = .010), having assistance (OR = 7.05; 95% CI, [2.54, 19.61]; *P* = .0002), and clear instructions (OR = 20.60; 95% CI, [6.87, 61.75]; *P* < .001).

**Conclusions:**

Findings indicate high receptivity to VR and highlight actionable levers, including usability, family engagement, assistance, and instructional supports, for implementation in oncology care.

## Introduction

Lung cancer constitutes a significant burden on the US healthcare system, with 218 893 new lung cancers reported in the United States in 2022, and 131 584 people in the United States died from lung cancer in 2023, based on the Centers for Disease Control’s statistics.^1^ Patients diagnosed with lung cancer often find it very complex and challenging to understand the available information on treatment options.^2^ Thus, it is essential to resort to shared decision-making (SDM) to empower patients’ choices and preferences.^2^

SDM could affect the ability to make an informed decision about potentially harmful treatment.^3^ It aims to increase patients’ information and control over treatment decisions, so that the decision better reflects the patients’ values and preferences.^3^ However, complex decision-making processes, such as treatment-related decisions, need prerequisites that patients often may lack, such as access to information and the ability to understand and synthesize the existing knowledge.^4^ To address this problem, decision aids are developed and introduced to clinical practice.^5^ Decision aids elucidate the specific decision at hand, outline available options, and present probable outcomes supported by current evidence, including benefits, harms, and uncertainties.^5^ Effective utilization of these aids makes patients more knowledgeable and clearer about their values and may improve risk perception.^2^^,^^3^^,^^6^ Decision aids for lung cancer treatment decision-making support are available in different formats, including printed and technology-based ones.^2^^,^^3^^,^^6^ Studies have shown that the effects of traditional decision aids’ formats on patient outcomes varied, but are generally weakly positive (eg, DAs improved patient satisfaction), with low evidence.^6^

In recent years, technology, including web-based platforms, mhealth, and interactive multimedia tools, has emerged as a critical facilitator of lung cancer treatment SDM. They have been shown to improve patients’ knowledge, reduce decisional conflict, and increase engagement in treatment choices.^7–9^ For example, web-based programs allow patients to review treatment information at their own pace, revisit content as needed, and prepare questions for their clinical visits.^8^ Mobile health tools and telehealth platforms further extend this support by providing real-time communication and remote delivery of educational content, which is especially valuable for patients in underserved or rural settings.^10^ Furthermore, technology-based decision aids can incorporate individual risk estimates and prognostic data, supporting more personalized and value-aligned decision-making.^9^ In the case of lung cancer, where treatment choices often involve balancing survival benefits, toxicity, and quality of life, these tools offer a structured way to help patients understand complex information and make decisions that reflect their preferences.^9^ A recent systematic review found that digital tools have been used to assist patients at different stages of the treatment journey, with demonstrated benefits in symptom management, reduced emotional distress, and improved patient-provider communication.^9^ However, the review also underscored persistent barriers, such as low digital literacy and unequal access to technology, which can limit the equitable implementation of these tools.^9^ These findings suggest that while technology can strengthen SDM in lung cancer care, innovative approaches are needed to address complexity, enhance patient engagement, and ensure accessibility across diverse patient populations.^9^ Virtual reality (VR) offers a promising extension of these technologies by providing an immersive and interactive environment for patient education.^11^

Unlike traditional digital tools, VR can simulate treatment experiences and present complex medical information in ways that are more intuitive and easier to understand.^11–13^ This immersive approach may help overcome barriers such as low health literacy or limited familiarity with medical terminology, both of which are identified as challenges in implementing conventional digital tools.^11^^,^^14–17^ By allowing patients to engage with information and experience realistic scenarios actively, VR-based interventions have the potential to improve comprehension, decrease anxiety, and promote more informed and value-concordant decision-making.^14–19^ Therefore, VR offers a new avenue to strengthen SDM in lung cancer care, addressing some limitations observed in earlier technology-driven methods.^18^^,^^19^ However, little attention has been given to studying patients’ acceptance of such technologies to support their SDM processes.^11^ In this study, we examine patients’ behavioral intentions to use VR for lung cancer decision-making support and identify predictors of this intention, guided by the Unified Theory of Acceptance and Use of Technology (UTAUT).

## Methods

### Study design

We conducted a cross-sectional survey-based perception and technology acceptance study to understand lung cancer patients’ anticipated willingness to use a virtual reality intervention for treatment preparedness and shared decision-making. At the time of data collection, a VR intervention had not yet been developed, and participants did not interact with a VR system before completing the survey. Instead, their responses were intended to inform the subsequent user-centered design and development of the EveryBreathMatters virtual reality platform. The questions used in this survey are presented in Table S1. Our study was reviewed and approved by the Institutional Review Board at Columbia University (IRB Protocol no. AAAV4003). Informed consent was obtained from all participants before data collection. The study adhered to the ethical principles outlined in the Declaration of Helsinki.

### Participants and recruitment

Participants were recruited between October 5th, 2024, and January 15th, 2025, using a convenience sampling strategy across multiple recruitment settings, including cancer centers, community clinics, and patient advocacy organizations. These recruitment sites were intentionally selected to increase access to patients from diverse socioeconomic backgrounds, including populations that may be underrepresented in oncology research. Recruitment was conducted through flyers, clinician referrals, advocacy organizations, and direct invitations to eligible patients. Eligible participants included lung cancer patients (aged 18 and older, diagnosed within the last 3 years, and who had begun treatment). Although recruitment sites were selected to improve sample diversity, participants were enrolled on a voluntary basis and therefore constitute a convenience sample rather than a representative sample of all lung cancer patients.

The participants’ rates are summarized in Table S2. Questions posed to patients centered on their experiences with treatment preparedness, barriers encountered, and preferences regarding the design and functionality of a VR tool for lung cancer treatment preparedness. Participants who consented could choose to complete the online survey. Survey links were sent via REDCap.

### Theoretical framework

This study is guided by the Unified Theory of Acceptance and Use of Technology (UTAUT), a widely validated framework for explaining individuals’ behavioral intention to adopt new technologies through 4 primary determinants: performance expectancy, effort expectancy, social influence, and facilitating conditions, together with moderators such as age, gender, experience, and voluntariness of use.^20–22^ UTAUT has demonstrated strong predictive validity across healthcare technologies, including telehealth and mobile health applications, particularly among older adults and individuals managing chronic diseases.^23–25^

In the context of our current study, these constructs were operationalized to reflect the experiences of lung cancer patients considering the use of VR to support treatment preparedness and shared decision-making. Performance expectancy represents patients’ beliefs that the VR system can improve their understanding of treatment options, increase preparedness for upcoming treatments, reduce uncertainty and anxiety, facilitate communication with clinicians, and ultimately support more informed treatment decisions. Effort expectancy reflects the anticipated ease of learning and interacting with the VR system despite common challenges experienced by lung cancer patients, including fatigue, dyspnea, cough, anxiety, or limited prior experience with immersive technologies. Social influence captures the extent to which encouragement from family members, caregivers, and healthcare professionals shapes patients’ willingness to use the intervention, recognizing that treatment decisions in oncology frequently involve close family participation. Facilitating conditions refer to the availability of technical assistance, clear instructional materials, onboarding support, and implementation resources that enable patients to comfortably integrate VR into their care experience.

Because participants had not yet used the EveryBreathMatters intervention, the present study focuses on behavioral intention to use VR rather than actual usage behavior, consistent with the UTAUT framework. Accordingly, the study examines how these constructs predict patients’ willingness to adopt VR as a decision-support tool for treatment preparedness. Behavioral intention was assessed under conditions of voluntary use rather than mandatory adoption. We recognize that, in future clinical implementation, recommendations from oncology providers or integration into routine clinical workflows may influence patients’ perceptions of voluntariness and subsequent technology adoption.

### Measures

We summarized the measures of this study in Table 1.

**Table 1.** Measures of the study.

Variable/construct	Exact survey question or instrument	Response options/score range	Variable construction and scoring	Use in analysis
Behavioral intention	“Are you willing to use such a tool to help with treatment decision-making and preparedness for treatment?” This aims to capture the intention to use VR rather than the actual system use.	Yes/no	Responses were coded as Yes = 1 and No = 0. No dichotomization was performed	Primary binary outcome in logistic regression
Performance expectancy	Two items assessed the degree to which participants expected VR to improve treatment preparedness: (1) motivation to start and engage in treatment, and (2) gaining knowledge needed to make and follow through on treatment decisions.	No/yes/neutral	Retained as categorical	Categorical predictor
Effort expectancy	Three items captured anticipated ease of use: (1) ease of navigating the VR tool (very easy to very difficult), (2) perceived added mental workload, and (3) perceived added physical workload.	No/yes/neutral	Retained as categorical	Categorical predictor
Social influence	Social influence was assessed with 3 items: (1) encouragement from healthcare providers to use technology for health purposes, (2) encouragement from family or friends, and (3) perceived importance of involving family and loved ones in the decision-making process.	No/yes/neutral	Retained as categorical	Categorical predictor
Facilitating conditions	Three items measured enabling factors: (1) encouragement if friends or family were also using the VR tool, (2) the helpfulness of having a specific person or group available to assist, and (3) the usefulness of clear instructions (written, video, or oral).	Yes/neutral/no	Retained as categorical	Categorical predictor
Quality of care (QOC)	How would you rate the quality of care received?	Good/neutral /bad	Responses were treated as an ordinal categorical variable.	Descriptive and inferential analyses
Health literacy	4-item BRIEF Health Literacy Screening Tool.^26^	Limited literacy/marginal literacy/adequate Literacy.	Items were scored on a 5-point scale and summed to a total score (range: 4-20), then categorized as per the BRIEF guidelines	Categorical variable
Digital literacy	8-item eHealth Literacy Scale (eHEALs).^27^ Items such as *"I know how to use the Internet to answer my questions about health"* were scored from 1 (*strongly disagree*) to 5 (*strongly agree*).	Scores ranged from 8 to 40, with higher scores indicating greater digital literacy.	Following prior studies, scores >24 were categorized as high digital literacy, while scores ≤24 were considered low digital literacy.	Continuous variable or categorized variable, depending on your analysis
VR familiarity	Are you familiar with VR?	Very familia/familiar/neutral/not familiar/not familiar at all	Responses were coded according to the original categories and analyzed as a categorical variable.	Covariate/descriptive variable
Family with technology	Do you use technology for health purposes (eg, patient portals, mobile health apps, and online records)?	Very familia/familiar/neutral/not familiar/not familiar at all	Responses were retained as categorical levels.	Categorical predictor
Demographic and clinical variables	Age, gender, race/ethnicity, education, income, cancer stage, and treatment status questions	Variable-specific response options	Age was analyzed as continuous; remaining variables were categorized according to the original survey response options or prespecified groupings.	Covariates and descriptive variables

### Data analysis

We first conducted descriptive analyses to summarize participant characteristics, technology use, familiarity with VR, and literacy levels. Continuous variables (age) were summarized using means and standard deviations, while categorical variables (eg, gender, education, and Quality of Care (QOC)) were summarized using frequencies and percentages.

To examine associations between demographic, literacy, and UTAUT factors and behavioral intention to use VR, we conducted Spearman rank correlation tests for continuous variables and Chi-square tests (or Fisher’s exact test where cell sizes were <5) for categorical variables.

Multivariable logistic regression was used to identify predictors of behavioral intention to use VR (yes/no), with UTAUT constructs as the primary independent variables. Odds ratios (ORs) and 95% confidence intervals (CIs) were reported. Regression models were adjusted for age and familiarity with technology. All tests were 2-tailed with a significance threshold of *P* < .05. Analyses were performed using Python v3.7 (New York, 2025). The dataset contained no missing values for the variables included in the analyses; therefore, all 200 participants were retained in the analyses.

## Results

### Descriptive statistics

Our study included *n* = 200 lung cancer patients. Table 2 summarizes their characteristics. Participants had a mean age of 62 years (SD = 15). Most of them were female (70%) and White (74%). Most participants reported having a degree beyond a bachelor’s (42%), while 27.5% had a high school education or less. Over half of the participants were unemployed (59%). Reported annual incomes varied, with 35% earning <$50 000. The sample included nearly half (44%) with government- or state-sponsored insurance, while only 1% were uninsured. Most of them had access to at least one regular provider (97.5%). At the time of data collection, 58.5% were still undergoing treatment, 39.5% had completed treatment, and 2% had discontinued treatment prematurely. Surgery (52%), chemotherapy (44%), and radiation (28%) were the most common treatment types, with smaller proportions receiving targeted therapy (19%), immunotherapy (11%), or other modalities (7.5%). The mean time since diagnosis was 27.7 months (SD = 25.2), with various stages of disease: 8% diagnosed at stage 4 and 31% at stage 1.

**Table 2.** Descriptive statistics of the study.

Variable		ALL	Do you use technology for health purposes (apps, online records, patient portals)?				Are you familiar with virtual reality?					
			Always	Often	Sometimes	Occasionally	Never	Not at all familiar	Somehow unfamiliar	Neutral	Familiar	Very familiar
		*N*	*N* = 88	*N* = 59	*N* = 35	*N* = 11	*N* = 6	*N* = 41	*N* = 32	*N* = 43	*N* = 73	*N* = 10
Age		62 ± 15	60 ± 15	61 ± 15	66 ± 14	68 ± 14	81 ± 7	69 ± 13	63 ± 14	60 ± 16	60 ± 16	53 ± 16
Gender	Female	140 (70%)	69 (78%)	41 (69%)	22 (63%)	4 (36%)	3 (50%)	27 (66%)	24 (75%)	31 (72%)	49 (67%)	8 (80%)
	Male	60 (30%)	19 (22%)	18 (31%)	13 (37%)	7 (64%)	3 (50%)	14 (34%)	8 (25%)	12 (28%)	24 (33%)	2 (20%)
Ethnicity	White	147 (74%)	70 (80%)	47 (80%)	19 (54%)	7 (64%)	4 (67%)	30 (73%)	25 (78%)	32 (74%)	53 (73%)	7 (70%)
	Black	13 (6.5%)	2 (2.3%)	5 (8.5%)	4 (11%)	1 (9.1%)	1 (17%)	4 (9.8%)	1 (3.1%)	2 (4.7%)	5 (6.8%)	1 (10%)
	Hispanic	14 (7.0%)	1 (1.1%)	3 (5.1%)	6 (17%)	3 (27%)	1 (17%)	7 (17%)	2 (6.3%)	3 (7.0%)	2 (2.7%)	0 (0%)
	Asian	17 (8.5%)	10 (11%)	3 (5.1%)	3 (8.6%)	0 (0%)	0 (0%)	0 (0%)	4 (13%)	3 (7.0%)	7 (9.6%)	2 (20%)
	Other	9 (4.5%)	5 (5.7%)	1 (1.7%)	3 (8.6%)	0 (0%)	0 (0%)	0 (0%)	0 (0%)	3 (7.0%)	6 (8.2%)	0 (0%)
Highest level of education	High school or less	55 (27.5%)	19 (22%)	13 (22%)	11 (31%)	7 (64%)	5 (83%)	14 (34%)	11 (34%)	10 (23%)	18 (25%)	2 (20%)
	Bachelor’s Degree	62 (31%)	25 (28%)	21 (36%)	14 (40%)	1 (9.1%)	1 (17%)	7 (17%)	10 (31%)	14 (33%)	27 (37%)	4 (40%)
	More than a Bachelor’s degree	83 (42%)	44 (50%)	25 (42%)	10 (29%)	3 (27%)	0 (0%)	20 (49%)	11 (34%)	19 (44%)	28 (38%)	4 (40%)
Employment status	Employed	82 (41%)	36 (41%)	26 (44%)	14 (40%)	5 (45%)	0 (0%)	10 (24%)	13 (41%)	13 (30%)	39 (53%)	6 (60%)
	Unemployed	118 (59%)	52 (59%)	33 (56%)	21 (60%)	6 (55%)	6 (100%)	31 (76%)	19 (59%)	30 (70%)	34 (47%)	4 (40%)
Annual income	< 50 000	70 (35%)	28 (32%)	17 (29%)	14 (40%)	6 (55%)	4 (67%)	18 (44%)	8 (25%)	18 (42%)	23 (32%)	2 (20%)
	50 000-99 999	44 (22%)	15 (17%)	17 (29%)	8 (23%)	3 (27%)	1 (17%)	9 (22%)	11 (34%)	8 (19%)	14 (19%)	2 (20%)
	100 000 and above	86 (43%)	45 (51%)	25 (42%)	13 (37%)	2 (18%)	1 (17%)	14 (34%)	13 (41%)	17 (40%)	36 (49%)	6 (60%)
Insurance status	No insurance	2 (1.0%)	1 (1.1%)	0 (0%)	0 (0%)	1 (9.1%)	0 (0%)	1 (2.4%)	0 (0%)	0 (0%)	0 (0%)	1 (10%)
	Government-sponsored or state-sponsored	88 (44%)	34 (39%)	28 (47%)	19 (54%)	6 (55%)	1 (17%)	25 (61%)	12 (38%)	20 (47%)	28 (38%)	3 (30%)
	Employer-sponsored insurance	66 (33%)	29 (33%)	24 (41%)	9 (26%)	2 (18%)	1 (17%)	6 (15%)	13 (41%)	11 (26%)	30 (41%)	5 (50%)
	Private insurance	33 (16.5%)	20 (23%)	5 (8.5%)	5 (14%)	2 (18%)	1 (17%)	5 (12%)	5 (16%)	11 (26%)	12 (16%)	0 (0%)
	Other	11 (5.5%)	4 (4.5%)	2 (3.4%)	2 (5.7%)	0 (0%)	3 (50%)	4 (9.8%)	2 (6.3%)	1 (2.3%)	3 (4.1%)	1 (10%)
Access to a regular healthcare provider	Yes	195 (97.5%)	86 (98%)	59 (100%)	32 (91%)	11 (100%)	6 (100%)	41 (100%)	32 (100%)	40 (93%)	71 (97%)	10 (100%)
	No/not sure	5 (2.5%)	2 (2.3%)	0 (0%)	3 (8.6%)	0 (0%)	0 (0%)	0 (0%)	0 (0%)	3 (7.0%)	2 (2.7%)	0 (0%)
Stage of diagnosis	Early Stage 1 and 2)	78 (39%)	29 (33%)	22 (37%)	16 (46%)	7 (64%)	4 (67%)	22 (54%)	11 (34%)	17 (40%)	25 (34%)	3 (30%)
	Late Stage 3 and 4)	95 (48%)	48 (55%)	31 (53%)	14 (40%)	1 (9.1%)	1 (17%)	11 (27%)	18 (56%)	22 (51%)	37 (51%)	7 (70%)
	Unknown/not staged	26 (13%)	11 (13%)	6 (10%)	5 (14%)	3 (27%)	1 (17%)	8 (20%)	3 (9.4%)	4 (9.3%)	11 (15%)	0 (0%)
Time sincediagnosis		27.7 ± 25.2	24 ± 24	34 ± 29	30 ± 23	19 ± 12	18 ± 21	28 ± 27	24 ± 26	26 ± 18	31 ± 28	22 ± 14
Health Literacy (14.32 ± 2.97)	Limited literacy	55 (28%)	19 (22%)	17 (29%)	14 (40%)	4 (36%)	1 (17%)	16 (39%)	14 (44%)	9 (21%)	15 (21%)	1 (10%)
	Marginal literacy	114 (57%)	54 (61%)	34 (58%)	18 (51%)	5 (45%)	3 (50%)	18 (44%)	15 (47%)	30 (70%)	45 (62%)	6 (60%)
	Adequate literacy	31 (16%)	15 (17%)	8 (14%)	3 (8.6%)	2 (18%)	2 (33%)	7 (17%)	3 (9.4%)	4 (9.3%)	13 (18%)	3 (30%)
Digital Literacy (30.68 ± 6.25)	High Digital literacy	177 (89%)	7 (8.0%)	5 (8.5%)	4 (11%)	3 (27%)	4 (67%)	7 (17%)	5 (16%)	5 (12%)	6 (8.2%)	0 (0%)
	Low digital literacy	23 (11%)	81 (92%)	54 (92%)	31 (89%)	8 (73%)	2 (33%)	34 (83%)	27 (84%)	38 (88%)	67 (92%)	10 (100%)

Digital literacy was generally high, with a mean score of 30.7 (SD = 6.3), and 89% of participants were classified as having high digital literacy. In contrast, health literacy was more limited, with a mean score of 14.3 (SD = 3.0); 57% had marginal literacy, 28% limited literacy, and only 16% adequate literacy.

### Technology acceptance based on demographics

#### Demographic characteristics

A total of *N* = 88 patients used technology for health purposes, and only *n* = 6 never used it. Most patients were not familiar with VR technology (*N* = 41 not at all familiar and *n* = 32 somewhat unfamiliar), and only *N* = 10 were very familiar with it. Patterns in technology use and VR familiarity varied across demographic groups (Table 2).

Older patients were less likely to use technology for health purposes (always: 60 ± 15; never: 81 ± 7). Women were more likely than men to be frequent technology users, with 78% of the “always use” group being female. Higher education was also associated with greater use: 50% of “always use” participants held a degree beyond a bachelor’s, compared to only 27% of those who never used technology. Similarly, participants with higher incomes (≥$100 000) were disproportionately represented in the “always use” category (51%).

Younger patients were more familiar with VR (Very familiar: 53 ± 16; Not at all familiar: 69 ± 13). Males were slightly more represented among those very familiar with VR (20%), but women remained the majority overall. Education showed a gradient: 40% of participants who were very familiar with VR had a bachelor’s degree or higher, whereas 83% of those who were not at all familiar had a high school education or less. Employment also played a role: more than half (53%) of participants who were familiar with VR worked full-time, compared with none in the “not at all familiar” group.

#### Socioeconomic factors

Patients with higher annual incomes (≥$100 000) were more likely to be familiar or very familiar with VR (49%-60%) than those with lower incomes (32%-42%). Insurance type also differed: participants with employer-sponsored insurance were better represented in the familiar/very familiar groups (41%-50%), while those with government/state-sponsored coverage were more common in the less familiar categories (61%).

#### Literacy

Health and digital literacy are strongly aligned with both technology and VR use. Participants with adequate health literacy were more concentrated in the familiar and very familiar VR groups (18%-30%) than in the “not at all familiar” group (9%). Likewise, high digital literacy was nearly universal among those who were very familiar with VR (100%) and significantly more common among frequent health technology users (92%) than among non-users (33%).

### Willingness to use VR for preparedness for lung cancer treatment

Of the 200 participants, 179 (89.5%) were willing to use VR for decision-making and treatment preparedness. The bivariate analyses examined differences between people who reported willingness to use VR for treatment preparedness and those who did not (Table 3). We found that patients willing to use VR were significantly younger (M = 60.8; SD = 15.3) than those unwilling (M = 73.2; SD = 10.8; *P < .*001).

**Table 3.** Descriptive statistics of the behavioral intention to use VR.

	Categories	No	Yes	*P* value
Age	Mean SD	73.19 (10.76)	60.78 (15.25)	<.001
Gender	Female	17	123	.364
	Male	4	56	
Race ethnicity	White	17	130	.645
	Black	1	12	
	Hispanic	2	12	
	Asian	0	17	
	Other	1	8	
Education	High School or Less	1	8	.989
	Bachelor’s Degree	5	41	
	More than a Bachelor’s Degree	15	130	
Employment	Employed	6	76	.395
	Unemployed	15	103	
Income	< 50 000	7	63	.059
	50 000-99 999	8	36	
	100 000 and above	6	80	
Insurance	No insurance	1	1	.165
	Government-sponsored or state-sponsored	11	77	
	Employer-sponsored insurance	6	60	
	Private insurance	1	32	
	Other	2	9	
Access to regular HCP	Yes	21	174	.849
	No/not sure	0	5	
Technology familiar	Always	5	84	.009
	Often	6	53	
	Sometimes	5	30	
	Occasionally	2	9	
	Never	3	3	
Health literacy	high literacy	14	141	.327
	low literacy	7	38	
BRIEF health literacy	Adequate	4	27	.656
	Limited	7	48	
	Marginal	10	104	

Additionally, familiarity with technology was significantly associated with willingness to use VR (*P = .*009). Those who always or often use health technology (*n* = 84 and *n* = 53, respectively) were more willing than the 3 participants in each group of “never users.”

The regression models adjusted for these 2 factors showed the following results (see Table 4).

**Table 4.** Regression analysis (UTAUT).

UTAUT construct	Variable	Categories	Behavioral intention			*P*
			Yes	No	OR [95% CI]	
Performance expectancy	Motivation to start and engage in treatment	No	35	18		<.001
		Yes	66	0	6.931 [4.06, 11.84]	
		Neutral	78	3	11.69 [3.49, 39.17]	
	Gaining knowledge needed to make and follow through on treatment decisions	No	25	15		<.001
		Yes	87	3	15.20 [4.39, 52.56]	
		Neutral	67	3	11.72 [3.37, 40.76]	
Effort expectancy	Ease of navigation	Difficult	23	12		<.001
		Easy	87	6	7.16 [2.50, 20.49]	
		Neutral	69	3	10.56 [2.95, 37.77]	
	Perceived added mental workload	Difficult	57	6		.312
		Easy	49	9	0.59 [0.20, 1.71]	
		Neutral	73	6	1.28 [0.41, 4.00]	
	Perceived added physical workload	Difficult	93	9		.732
		Easy	29	4	0.67 [0.20, 2.20]	
		Neutral	57	8	0.69 [0.26, 1.83]	
Social influence	Encouragement from HCP	No	44	8		.283
		Yes	135	13	1.92 [0.76, 4.82]	
	Encouragement from family and friends	No	74	15		.016
		Yes	105	6	3.38 [1.29, 8.84]	
	Importance of involving family and loved ones	No	58	13		.015
		Yes	121	8	3.30 [1.32, 8.22]	
Facilitating conditions	Family and friends were also using the tool	No	46	12		.01
		Yes	86	5	4.23 [1.46, 12.26]	
		Neutral	47	4	2.84 [0.90, 8.97]	
	Having specific people to assist in the process	No	18	9		.0002
		Yes	130	9	7.05 [2.54, 19.61]	
		Neutral	31	3	4.62 [1.19, 17.89]	
	Clear instructions	No	11	11		<.001
		Yes	154	7	20.60 [6.87, 61.75]	
		Neutral	14	3	4.14 [1.00, 17.23]	

#### Performance expectancy

Both aspects of performance expectancy were strongly predictive of intention. Patients who believed VR would motivate them to start and engage in treatment were significantly more likely to report willingness to engage in treatment (*P < .*001). Compared with those who answered “no,” the odds of willingness were nearly 7 times higher for those responding “yes” (OR = 6.93; 95% CI, [4.06, 11.84]) and over 11 times higher for those responding “neutral” (OR = 11.69; 95% CI, [3.49, 39.17]). Similarly, perceiving VR as helpful in gaining knowledge for decision-making was highly predictive (*P < .*001), with odds of willingness 15.2 times higher among those responding “yes” (95% CI, [4.39, 52.56]) and 11.7 times higher among “neutral” respondents (95% CI, [3.37, 40.76]).

#### Effort expectancy

Ease of navigation was significantly associated with willingness (*P < .*001). Compared with those who found VR “difficult,” participants who perceived it as “easy” (OR = 7.16; 95% CI, [2.50, 20.49]) or “neutral” (OR = 10.56; 95% CI, [2.95, 37.77]) were more likely to report an intention to use. In contrast, perceived mental workload (*P = .*312) and physical workload (*P = .*732) were not significantly associated with willingness.

#### Social influence

Family and social encouragement emerged as strong predictors. Participants who reported encouragement from family and friends were over 3 times more likely to be willing (OR = 3.38; 95% CI, [1.29, 8.84]; *P = .*016). Similarly, those who endorsed the importance of involving family members and loved ones in decision-making were over 3 times more likely to report willingness (OR = 3.30; 95% CI, [1.32, 8.22]; *P = .*015). Encouragement from healthcare providers was not statistically significant (*P = .*283).

#### Facilitating conditions

Several facilitating factors were significantly associated with intention. Willingness was more than 4 times higher among participants who believed VR would be encouraged if family or friends were using it (OR = 4.23; 95% CI, [1.46, 12.26]; *P = .*010). Having a specific person available to assist with technology increased the odds 7-fold (OR = 7.05; 95% CI, [2.54, 19.61]; *P = .*0002). Clear instructions were the strongest predictor, with participants who endorsed their usefulness being over 20 times more likely to intend to use VR (OR = 20.60; 95% CI, [6.87, 61.75]; *P < .*001).

Overall, performance expectancy, ease of navigation, family/social encouragement, facilitating support, and clear instructions were the most significant predictors of behavioral intention. In contrast, perceptions of workload and healthcare provider encouragement were not significantly associated.

## Discussion

This study aimed to explore lung cancer patients’ perceptions of VR’s role in supporting their shared decision-making regarding treatment. The study involved 200 patients, 179 (89.5%) of whom were highly willing to use VR for treatment preparedness support, with willing participants significantly younger than those who were not willing (mean 60.8 vs 73.2 years). This finding expands on an extensive survey study showing that cancer patients accept the use of VR for emotional regulation by extending the acceptability to different clinical purposes, decision-making, and among a specific population, lung cancer patients.^13^ This high level of behavioral intention suggests strong patient receptivity and supports progression to subsequent implementation studies evaluating actual VR uptake, sustained use, usability, and clinical effectiveness. This finding also highlights the need to develop age-friendly VR tools that accommodate patients’ age-based needs.

Guided by the UTAUT, we found that Performance Expectancy, Effort Expectancy, Social Influence, and Facilitating Conditions predicted intention to use VR for treatment preparedness support and decision-making. Our results reinforce the use of the UTAUT in oncology and for VR adoption.

We found that both aspects of performance expectancy were strong predictors of the intention to use VR. Patients who believed VR would motivate them to start and engage in treatment, and those who thought it would help them gain the knowledge needed to engage in treatment, were more likely to report willingness. These findings coincide with the core of the UTAUT model, where the perceived usefulness (performance expectancy) emerges as the strongest determinant of behavioral intention.^23^ Studies on other types of technology tools (mhealth, telehealth, etc.) show the same pattern among cancer patients.^28^^,^^29^ However, there were mixed results for traditional decision aids in oncology, with evidence on motivation and preparedness outcomes being weak.^6^ Our findings suggest that immersive VR may overcome these limitations by coupling intuitive visualization with motivational reinforcement.

We also found that ease of navigation in VR was a significant predictor of intention to use the tool. Participants who found VR easy or were neutral about it were 7 to 10 times more likely to report willingness compared to those who found it challenging. This result coincides with the UTAUT literature, which confirms that effort expectancy is a consistent determinant of adoption in digital health tools, but usually less strong than performance expectancy.^20^

It is also noteworthy that mental and physical workload were not associated with the willingness, which differs from previous literature on the use of technology that generally reports that cognitive burden is a barrier to adoption.^17^ This can be explained by the engaging nature of VR environments, compared with other tools, which offsets the cognitive effort required. Researchers and clinics introducing VR tools to cancer patients should highlight their ease of use to ease pre-use anxiety, rather than worrying too much about cognitive or physical strain. Future studies should also explore whether VR-related workload tolerance remains consistent across various demographic groups.

Our results showed that family and social encouragement were significantly associated with the intention to use VR. Patients encouraged by family or friends were more than th3ree times as likely to report willingness (OR = 3.38). Similarly, those who thought family involvement in decision-making was important were also over 3 times more likely to plan to use VR (OR = 3.30). Our results align with studies showing the role of family and peer encouragement in increasing technology use, especially in cancer care.^23^^,^^30^ On the other hand, encouragement from healthcare providers was not significantly linked to willingness, which differs from previous studies reporting clinician endorsement as a strong motivator of technology adoption.^31^^,^^32^ Overall, these results expand on the collectivist and shared decision-making work in cancer care, showing that VR adoption is socially shaped not only by individual perceptions but also by the degree to which patients perceive family participation as central to treatment decisions.^33^ Oncology teams should recognize the importance of caregivers as co-decision makers, and training on VR adoption should be extended to both patients and families.

When assessing possible facilitating conditions, we identified several associated factors. Patients who believed they would be encouraged if family and friends were also using the tool were 4 times more likely to report willingness. This finding is in alignment with the observational learning and social contagion theories, in which seeing others engage with a technology reduces uncertainty among users and builds more trust in its utility.^34^^,^^35^ This result suggests a network effect in which adoption could spread more effectively through social groups and communities rather than isolated individuals.^36^

In addition, we found that patients were 7 times more likely to intend to use VR if they believed someone would be available to assist them with the tool. In cancer contexts, similar findings exist for telehealth and digital symptom management platforms, where support availability was a determinant of sustained use.^37^ However, our study is among the first to explore this association in the context of VR use. Another result that highlights the role of assisting patients in promoting their technology use is that patients who endorsed the usefulness of clear instructions were significantly more likely to intend to use VR for treatment preparedness, which was the strongest predictor across all UTAUT components. VR rollouts in oncology should integrate support roles (eg, navigators, trained family members, or peer volunteers) and instructional scaffolding (eg, pictorial and interactive tutorials, and instructions in multiple formats) into adoption strategies.

Some limitations to our study are worth acknowledging. First, the cross-sectional design limits causal inference. In addition, although multiple studies use intention to use as a proxy for use, this measure may overestimate actual VR uptake. Additionally, the convenience sample (high digital literacy, mostly White, lung cancer only) may constrain the generalizability of our findings. Also, self-reported measures can introduce common-method bias; however, our use of strong, theory-consistent patterns across multiple UTAUT constructs aimed to reduce this concern. Future work should include behavioral usage logs, clinician-level variables, and site-level factors.

## Conclusions

Our study examined lung cancer patients’ intentions to use virtual reality (VR) for treatment preparedness and shared decision-making, guided by the UTAUT framework. Findings demonstrate strong patient receptivity and highlight key predictors of adoption, providing evidence that VR holds promise as an innovative tool to enhance patient engagement and support in oncology care.

## Supplementary Material

ooag178_Supplementary_Data

## Data Availability

The data can be made available upon reasonable request from the corresponding author.

## Code availability

The code can be provided upon request from the corresponding author.
