# Supplementary material for: Predictors of lung cancer patients’ behavioral intention to use virtual reality for treatment preparedness and shared decision-making: a Unified Theory of Acceptance and Use of Technology-based perception study
Source: JAMIA Open. 2026 Sep 25;9(5):ooag178. doi: 10.1093/jamiaopen/ooag178 (PMC13613060; doi:10.1093/jamiaopen/ooag178)
Supplement: Supplementary file 1 [file ooag178_supplementary_data.docx]

**APPENDIX A. Questions used in the survey.**

| **Domain** | **Item (verbatim/construct)** | **Response Options / Scale** | **Notes** |
| --- | --- | --- | --- |
| **Quality of Care (QOC)** | Perceived quality of cancer care received | Good; Neutral; Bad | Single item; 3-point Likert |
| **Demographics** | Age | Open numeric | Years |
|  | Gender | Female; Male; Non-binary; Other |  |
|  | Race/Ethnicity | White; Black; Hispanic; American Indian; Asian; Other |  |
|  | Highest education | Less than high school; High school; Some college; Bachelor's; > Bachelor's |  |
|  | Employment status | Full-time; Part-time; Unemployed/Looking; Unemployed/Not working; Other | |
|  | Annual income | <$25k; $25–49k; $50–74k; $75–99k; $100–149k; ≥$150k |  |
|  | Insurance coverage | None; Gov’t/state; Employer-sponsored; Private; Other |  |
|  | Stage of diagnosis | I; II; III; IV; Unknown/Not staged | Collapsed in results too early (I–II) vs late (III–IV) when needed |
| **Technology Use & Familiarity** | "Do you use technology for health purposes (apps, online records, portals)?" | Always; Often; Sometimes; Occasionally; Never | Categorical frequency |
|  | "Are you familiar with Virtual Reality (VR)?" | Not at all familiar; Somehow unfamiliar; Neutral; Familiar; Very familiar | 5-point familiarity scale |
| **Behavioral Intention (UTAUT)** | "Are you willing to use such a tool to help with treatment decision-making and preparedness for treatment?" | Yes; No | Primary outcome |
| **Performance Expectancy (UTAUT)** | "To what extent will VR motivate you to start and engage in treatment?" | Very much; Somehow; Neutral; Not much; Not at all | 5-point Likert |
|  | "To what extent will VR help you gain the knowledge needed to decide and follow through on treatment?" | Very much; Somehow; Neutral; Not much; Not at all | 5-point Likert |
| **Effort Expectancy (UTAUT)** | "Do you think it will be easy for you to navigate such a tool?" | Very easy; Easy; Neutral; Difficult; Very difficult | 5-point ease/difficulty |
|  | "To what extent will the tool add to your mental workload?" | Very much; Somehow; Neutral; Not much; Not at all | 5-point Likert |
|  | "To what extent will the tool add to your physical workload?" | Very much; Somehow; Neutral; Not much; Not at all | 5-point Likert |
| **Social Influence (UTAUT)** | Encouragement from healthcare providers to use health technology | Yes, often; Yes, one/few times; No, never; Don’t recall | Categorical frequency |
|  | Encouragement from family/friends to use health technology | Yes, often; Yes, one/few times; No, never; Don’t recall | Categorical frequency |
|  | "It is important to involve family/loved ones in the decision-making process." | Totally agree; Agree; Neutral; Disagree; Totally disagree | 5-point agreement |
| **Facilitating Conditions (UTAUT)** | "Would you feel more encouraged if friends/family/peers were also using the tool?" | Very much; Somehow; Neutral; Not much; Not at all | 5-point Likert |
|  | "How helpful would it be to have a specific person/group available to assist?" | Very much; Somehow; Neutral; Not much; Not at all | 5-point Likert |
|  | "How helpful would clear instructions (written/video/oral) be?" | Very much; Somehow; Neutral; Not much; Not at all | 5-point Likert |
| **Digital Literacy (eHEALS)** | 8 items (e.g., "I know how to use the Internet to answer my questions about health.”) | 1–5: Strongly disagree → Strongly agree | Sum 8–40; categorize: >24 = High, ≤24 = Low |
| **Health Literacy (BRIEF)** | 4 items (help reading materials; problems understanding written info; problems understanding what is told; confidence filling forms) | 1–5 scale per item (per BRIEF guidance) | Sum 4–20; categorize: 4–12 Limited; 13–16 Marginal; 17–20 Adequate |

**APPENDIX B. Recruitment sources for the different participants**

| **Method of Recruitment** | **Rate of participants** |
| --- | --- |
| Social Media (Cancer support groups) | 40% |
| Patient community groups (Organizations and support groups) | 20% |
| Healthcare provider referral | 20% |
| RecruitMe platform | 10% |
| Hospital flyer | 3% |
| Other | 7% |

**APPENDIX C. DESCRIPTIVE STATISTICS OF THE STUDY**

| **Variable** | | **ALL** | **Do you use technology for health purposes (apps, online records, patient portals)?** | | | | **Are you familiar with Virtual Reality?** | | | | | |
| --- | --- | --- | --- | --- | --- | --- | --- | --- | --- | --- | --- | --- |
|  |  |  | **Always** | **Often** | **Sometimes** | **Occasionally** | **Never** | **Not at**  **all familiar** | **Somehow unfamiliar** | **Neutral** | **Familiar** | **Very familiar** |
|  |  | **N** | N = 88*^1^* | N = 59*^1^* | N = 35*^1^* | N = 11*^1^* | N = 6*^1^* | N = 41*^1^* | N = 32*^1^* | N = 43*^1^* | N = 73*^1^* | N = 10*^1^* |
| **Age** | | **62 ± 15** | 60 ± 15 | 61 ± 15 | 66 ± 14 | 68 ± 14 | 81 ± 7 | 69 ± 13 | 63 ± 14 | 60 ± 16 | 60 ± 16 | 53 ± 16 |
| **Gender** | Female | 140 (70%) | 69 (78%) | 41 (69%) | 22 (63%) | 4 (36%) | 3 (50%) | 27 (66%) | 24 (75%) | 31 (72%) | 49 (67%) | 8 (80%) |
|  | Male | 60 (30%) | 19 (22%) | 18 (31%) | 13 (37%) | 7 (64%) | 3 (50%) | 14 (34%) | 8 (25%) | 12 (28%) | 24 (33%) | 2 (20%) |
| **Ethnicity** | White | 147 (74%) | 70 (80%) | 47 (80%) | 19 (54%) | 7 (64%) | 4 (67%) | 30 (73%) | 25 (78%) | 32 (74%) | 53 (73%) | 7 (70%) |
|  | Black | 13 (6.5%) | 2 (2.3%) | 5 (8.5%) | 4 (11%) | 1 (9.1%) | 1 (17%) | 4 (9.8%) | 1 (3.1%) | 2 (4.7%) | 5 (6.8%) | 1 (10%) |
|  | Hispanic | 14 (7.0%) | 1 (1.1%) | 3 (5.1%) | 6 (17%) | 3 (27%) | 1 (17%) | 7 (17%) | 2 (6.3%) | 3 (7.0%) | 2 (2.7%) | 0 (0%) |
|  | Asian | 17 (8.5%) | 10 (11%) | 3 (5.1%) | 3 (8.6%) | 0 (0%) | 0 (0%) | 0 (0%) | 4 (13%) | 3 (7.0%) | 7 (9.6%) | 2 (20%) |
|  | Other | 9 (4.5%) | 5 (5.7%) | 1 (1.7%) | 3 (8.6%) | 0 (0%) | 0 (0%) | 0 (0%) | 0 (0%) | 3 (7.0%) | 6 (8.2%) | 0 (0%) |
| **Highest Level of Education** | High School or Less | 55 (27.5%) | 19 (22%) | 13 (22%) | 11 (31%) | 7 (64%) | 5 (83%) | 14 (34%) | 11 (34%) | 10 (23%) | 18 (25%) | 2 (20%) |
|  | Bachelor's Degree | 62 (31%) | 25 (28%) | 21 (36%) | 14 (40%) | 1 (9.1%) | 1 (17%) | 7 (17%) | 10 (31%) | 14 (33%) | 27 (37%) | 4 (40%) |
|  | More than a Bachelor's Degree | 83 (42%) | 44 (50%) | 25 (42%) | 10 (29%) | 3 (27%) | 0 (0%) | 20 (49%) | 11 (34%) | 19 (44%) | 28 (38%) | 4 (40%) |
| **Employment Status** | Employed | 82 (41%) | 36 (41%) | 26 (44%) | 14 (40%) | 5 (45%) | 0 (0%) | 10 (24%) | 13 (41%) | 13 (30%) | 39 (53%) | 6 (60%) |
|  | Unemployed | 118 (59%) | 52 (59%) | 33 (56%) | 21 (60%) | 6 (55%) | 6 (100%) | 31 (76%) | 19 (59%) | 30 (70%) | 34 (47%) | 4 (40%) |
| **Annual Income** | < 50,000 | 70 (35%) | 28 (32%) | 17 (29%) | 14 (40%) | 6 (55%) | 4 (67%) | 18 (44%) | 8 (25%) | 18 (42%) | 23 (32%) | 2 (20%) |
|  | 50,000 - 99,999 | 44 (22%) | 15 (17%) | 17 (29%) | 8 (23%) | 3 (27%) | 1 (17%) | 9 (22%) | 11 (34%) | 8 (19%) | 14 (19%) | 2 (20%) |
|  | 100,000 and above | 86 (43%) | 45 (51%) | 25 (42%) | 13 (37%) | 2 (18%) | 1 (17%) | 14 (34%) | 13 (41%) | 17 (40%) | 36 (49%) | 6 (60%) |
| **Insurance Status** | No insurance | 2 (1.0%) | 1 (1.1%) | 0 (0%) | 0 (0%) | 1 (9.1%) | 0 (0%) | 1 (2.4%) | 0 (0%) | 0 (0%) | 0 (0%) | 1 (10%) |
|  | Government-sponsored or state-sponsored | 88 (44%) | 34 (39%) | 28 (47%) | 19 (54%) | 6 (55%) | 1 (17%) | 25 (61%) | 12 (38%) | 20 (47%) | 28 (38%) | 3 (30%) |
|  | Employer-sponsored insurance | 66 (33%) | 29 (33%) | 24 (41%) | 9 (26%) | 2 (18%) | 1 (17%) | 6 (15%) | 13 (41%) | 11 (26%) | 30 (41%) | 5 (50%) |
|  | Private Insurance | 33 (16.5%) | 20 (23%) | 5 (8.5%) | 5 (14%) | 2 (18%) | 1 (17%) | 5 (12%) | 5 (16%) | 11 (26%) | 12 (16%) | 0 (0%) |
|  | Other | 11 (5.5%) | 4 (4.5%) | 2 (3.4%) | 2 (5.7%) | 0 (0%) | 3 (50%) | 4 (9.8%) | 2 (6.3%) | 1 (2.3%) | 3 (4.1%) | 1 (10%) |
| **Access to a Regular Healthcare Provider** | Yes | 195 (97.5%) | 86 (98%) | 59 (100%) | 32 (91%) | 11 (100%) | 6 (100%) | 41 (100%) | 32 (100%) | 40 (93%) | 71 (97%) | 10 (100%) |
|  | No/Not sure | 5 (2.5%) | 2 (2.3%) | 0 (0%) | 3 (8.6%) | 0 (0%) | 0 (0%) | 0 (0%) | 0 (0%) | 3 (7.0%) | 2 (2.7%) | 0 (0%) |
| **Stage of Diagnosis** | Early Stage 1 and 2) | 78 (39%) | 29 (33%) | 22 (37%) | 16 (46%) | 7 (64%) | 4 (67%) | 22 (54%) | 11 (34%) | 17 (40%) | 25 (34%) | 3 (30%) |
|  | Late Stage 3 and 4) | 95 (48%) | 48 (55%) | 31 (53%) | 14 (40%) | 1 (9.1%) | 1 (17%) | 11 (27%) | 18 (56%) | 22 (51%) | 37 (51%) | 7 (70%) |
|  | Unknown/Not staged | 26 (13%) | 11 (13%) | 6 (10%) | 5 (14%) | 3 (27%) | 1 (17%) | 8 (20%) | 3 (9.4%) | 4 (9.3%) | 11 (15%) | 0 (0%) |
| **Time since Diagnosis** | | **27.7 ± 25.2** | 24 ± 24 | 34 ± 29 | 30 ± 23 | 19 ± 12 | 18 ± 21 | 28 ± 27 | 24 ± 26 | 26 ± 18 | 31 ± 28 | 22 ± 14 |
| **Health Literacy (14.32 ± 2.97)** | Limited Literacy | 55 (28%) | 19 (22%) | 17 (29%) | 14 (40%) | 4 (36%) | 1 (17%) | 16 (39%) | 14 (44%) | 9 (21%) | 15 (21%) | 1 (10%) |
|  | Marginal Literacy | 114 (57%) | 54 (61%) | 34 (58%) | 18 (51%) | 5 (45%) | 3 (50%) | 18 (44%) | 15 (47%) | 30 (70%) | 45 (62%) | 6 (60%) |
|  | Adequate Literacy | 31 (16%) | 15 (17%) | 8 (14%) | 3 (8.6%) | 2 (18%) | 2 (33%) | 7 (17%) | 3 (9.4%) | 4 (9.3%) | 13 (18%) | 3 (30%) |
| **Digital Literacy (30.68 ± 6.25)** | High Digital Literacy | 177 (89%) | 7 (8.0%) | 5 (8.5%) | 4 (11%) | 3 (27%) | 4 (67%) | 7 (17%) | 5 (16%) | 5 (12%) | 6 (8.2%) | 0 (0%) |
|  | Low Digital Literacy | 23(11%) | 81 (92%) | 54 (92%) | 31 (89%) | 8 (73%) | 2 (33%) | 34 (83%) | 27 (84%) | 38 (88%) | 67 (92%) | 10 (100%) |

**APPENDIX D. REGRESSION ANALYSIS (UTAUT)**

| **UTAUT Construct** | **Variable** | **Categories** | **Behavioral intention** | | |  |  |
| --- | --- | --- | --- | --- | --- | --- | --- |
|  |  |  |  |  |  |  |  |
|  |  |  | **Yes** | **No** | **OR [95%CI]** |  |  |
| **Performance Expectancy** | **motivation to start and engage in treatment** | **No** | 35 | 18 |  | **<.001** |  |
|  |  | **Yes** | 66 | 0 | 6.931 [4.06, 11.84] |  |  |
|  |  | **Neutral** | 78 | 3 | 11.69 [3.49, 39.17] |  |  |
|  | **gaining knowledge needed to make and follow through on treatment decisions** | **No** | 25 | 15 |  | **<.001** |  |
|  |  | **Yes** | 87 | 3 | 15.20 [4.39, 52.56] |  |  |
|  |  | **Neutral** | 67 | 3 | 11.72 [3.37, 40.76] |  |  |
| **Effort Expectancy** | **Ease of navigation** | **Difficult** | 23 | 12 |  | **<.001** |  |
|  |  | **Easy** | 87 | 6 | 7.16 [2.50, 20.49] |  |  |
|  |  | **Neutral** | 69 | 3 | 10.56 [2.95, 37.77] |  |  |
|  | Perceived added mental workload | **Difficult** | 57 | 6 |  | **0.312** |  |
|  |  | **Easy** | 49 | 9 | 0.59 [0.20, 1.71] |  |  |
|  |  | **Neutral** | 73 | 6 | 1.28 [0.41, 4.00] |  |  |
|  | Perceived added physical workload | **Difficult** | 93 | 9 |  | **0.732** |  |
|  |  | **Easy** | 29 | 4 | 0.67 [0.20, 2.20] |  |  |
|  |  | **Neutral** | 57 | 8 | 0.69 [0.26, 1.83] |  |  |
| **Social Influence** | Encouragement from HCP | **No** | 44 | 8 |  | **0.283** |  |
|  |  | **Yes** | 135 | 13 | 1.92 [0.76, 4.82] |  |  |
|  | **Encouragement from family and friends** | **No** | 74 | 15 |  | **0.016** |  |
|  |  | **Yes** | 105 | 6 | 3.38 [1.29, 8.84] |  |  |
|  | **Importance of involving family and loved ones** | **No** | 58 | 13 |  | **0.015** |  |
|  |  | **Yes** | 121 | 8 | 3.30 [1.32, 8.22] |  |  |
| **Facilitating conditions** | **Family and friends were also using the tool** | **No** | 46 | 12 |  | **0.01** |  |
|  |  | **Yes** | 86 | 5 | 4.23 [1.46, 12.26] |  |  |
|  |  | **Neutral** | 47 | 4 | 2.84 [0.90, 8.97] |  |  |
|  | **Having specific people to assist in the process** | **No** | 18 | 9 |  | **0.0002** |  |
|  |  | **Yes** | 130 | 9 | 7.05 [2.54, 19.61] |  |  |
|  |  | **Neutral** | 31 | 3 | 4.62 [1.19, 17.89] |  |  |
|  | **Clear Instructions** | **No** | 11 | 11 |  | **<.001** |  |
|  |  | **Yes** | 154 | 7 | 20.60 [6.87, 61.75] |  |  |
|  |  | **Neutral** | 14 | 3 | 4.14 [1.00, 17.23] |  |  |
